# Implications of Insulin-Like Growth Factor-1 in Skeletal Muscle and Various Diseases

**DOI:** 10.3390/cells9081773

**Published:** 2020-07-24

**Authors:** Syed Sayeed Ahmad, Khurshid Ahmad, Eun Ju Lee, Yong-Ho Lee, Inho Choi

**Affiliations:** 1 Department of Medical Biotechnology, Yeungnam University, Gyeongsan 38541, Korea; sayeedahmad4@gmail.com (S.S.A.); ahmadkhursheed2008@gmail.com (K.A.); gorapadoc0315@hanmail.net (E.J.L.); 2 Research Institute of Cell Culture, Yeungnam University, Gyeongsan 38541, Korea; 3 Department of Biomedical Science, Daegu Catholic University, Gyeongsan 38430, Korea

**Keywords:** skeletal muscle, IGF-1, MSCs, myogenesis

## Abstract

Skeletal muscle is an essential tissue that attaches to bones and facilitates body movements. Insulin-like growth factor-1 (IGF-1) is a hormone found in blood that plays an important role in skeletal myogenesis and is importantly associated with muscle mass entity, strength development, and degeneration and increases the proliferative capacity of muscle satellite cells (MSCs). IGF-1R is an IGF-1 receptor with a transmembrane location that activates PI3K/Akt signaling and possesses tyrosine kinase activity, and its expression is significant in terms of myoblast proliferation and normal muscle mass maintenance. IGF-1 synthesis is elevated in MSCs of injured muscles and stimulates MSCs proliferation and myogenic differentiation. Mechanical loading also affects skeletal muscle production by IGF-1, and low IGF-1 levels are associated with low handgrip strength and poor physical performance. IGF-1 is potentially useful in the management of Duchenne muscular dystrophy, muscle atrophy, and promotes neurite development. This review highlights the role of IGF-1 in skeletal muscle, its importance during myogenesis, and its involvement in different disease conditions.

## 1. Introduction

Muscles attached to the bone are referred to as skeletal muscle (SM) and account for 30–50% of body weight and are responsible for skeletal movement. In the human body, SM is one of the most plastic and dynamic tissue and utilizes up to 50–75% of all body proteins [1,2]. SM cell proliferation and differentiation are vitally required for appropriate SM development throughout embryogenesis and for postnatal SM regeneration that is essential for muscle healing after injury [3]. In multicellular organisms, cell generation in all tissues is under the control of a network of tissue-specific regulators termed growth factors (GFs). GFs are low molecular weight peptides that are active during cell proliferation and differentiation [4,5], migration, and apoptosis, and play a significant role in managing growth signal responses throughout development [6]. GFs have been reported in blood vessels and epithelial, lymphoid, neural, muscle, lymphatic, erythroid, myeloid, and hepatic systems, and few GFs and cytokines are produced in each tissue [7]. GFs also regulate cellular responses during wound healing and act as endogenous signaling molecules [8]. Wound healing is a multifaceted physiological process that involves interplay between numerous cell types, GFs, extracellular matrix (ECM) constituents, and proteinases [9].

ECM is well known to preserve SM integrity and participates throughout myogenesis. Our group has explored the contributions made by several ECM components, e.g., fibromodulin [10,11,12], dermatopontin [2], and matrix gla protein [13], during myogenesis. In recent decades, the number of cases of debilitating injury has increased, and the treatment of individuals suffering from different chronic injuries incurs substantial costs, especially in the United States and Europe [14,15,16]. At each stage of healing, specific arrangements of cytokines and GFs must cooperate with their respective receptors and ECM constituents at their target locations [17,18].

GFs play a substantial role in tissue recovery as well as in the regulation of diverse cellular processes and act as signaling molecules between cells. Because of their instabilities and soluble natures, developments are required to enable their therapeutic use [19]. GF delivery has been a theme of augmented recent research attention owing to the controlled and targeted drug delivery in addition to the development of recombinant DNA methods that have enabled GFs creation [20,21,22]. Heparin, a profoundly sulfated glycosaminoglycan, has been used to facilitate the local delivery of GFs from different matrices (e.g., microcapsules [23]), as it binds and potentiates the activities of GFs. Specifically, heparin has been shown to prevent the deactivation of GFs [21,24], enhance their interactions with receptors [25], increase GF loading into delivery vehicles [26], and facilitate the long-terms releases of GFs [26,27].

**Figure 1:**
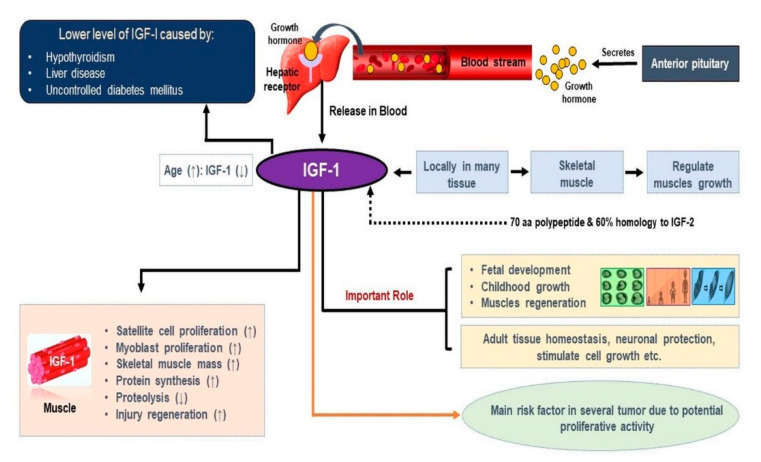
Role of insulin-like growth factor-1 (IGF-1) in skeletal muscle. IGF-1 is responsible for fetal development, child growth, and muscle regeneration, and elevated IGF-1 levels are required for muscle satellite cell (MSC) and myoblast proliferation, postinjury regeneration, and the increase of skeletal mass.

Components of the endocrine system, such as growth hormone (GH), insulin-like growth factor-1 (IGF-1), and androgens, are the foremost regulators of muscle metabolism. These endocrine components have substantial impacts on muscle and act as anabolic factors and significant regulators of muscle mass [28]. IGF-1 is a 70 aa polypeptide with autocrine, paracrine, and endocrine properties, and shares a ~60% similarity with IGF-2 and a 50% similarity with proinsulin structures [29]. The actions of IGF-1 and 2 are mainly facilitated by type 1 receptors. Insulin-like growth factor type 1 receptor (IGF-1R) is required for cell growth and development and to maintain the cell cycle. IGF-1 and IGF-2 are also known as mitogenic peptides that show homology with each other and with insulin [30,31,32,33]. IGF-1 is considered to play key roles in fetal development and growth up to adolescence, and in the maintenance of homeostasis in adult tissues by regulating cell proliferation, differentiation, and survival (Figure 1). It has also been reported IGF-1 has atheroprotective, neuroprotective, and insulin-like effects and that it regulates skeletal muscle metabolism and regeneration [34]. Physiological maintenance of SM requires injury or stretch stimulation, which prompts IGF-1 expression [35]. The supplementation of pro-IGF-2 could be one of the most effective therapeutic approaches for muscle injury in elderly people [36].

IGF-1 mRNA gives rise to three proforms, IGF-1Ea, IGF-1Eb, and IGF-1Ec, which yield three different C-terminal extensions called Ea, Eb, and Ec peptides [37]. IGF-1Ea and IGF-1Eb are necessary for the initiation of myogenesis in mice, but the loss of IGF-1Ea is related to greater reductions in myogenesis than IGF-1Eb [38]. Interestingly, IGF-1Ea is upregulated by a single ramp stretch of one hour but reduced by repeated cyclical stretches, whereas IGF-1Eb is upregulated by cycling stacking [39]. At the point when the typical strain and stretch are not set up, the IGF-1 signaling pathway turns into deactivated and prompts muscle atrophy, as appeared in astronauts working in the microgravity environment [40]. IGF-1 is synthesized and released from the liver along with some other tissue such as muscle, heart, adipose tissue, brain, and pancreatic β-cell [41]. IGF1 proforms can induce breast cancer cell proliferation through its receptor [42]. IGF-1 is the main regulator of growth and metabolism in mammals [31,43]. Circulating IGF-1 is controlled by members of the IGF binding protein family (IGFBP-1~6) and acid-labile subunit (ALS). GH, insulin, and nutritional status are responsible for the secretion of IGF-1 [44,45]. The maintenance of hypertrophic phenotype by IGF-1Ea involves also the activation of AMPK pathways, a factor involved in the maintenance of whole-body energy balance and an “energy sensor” controlling glucose and lipid metabolism [46]. Either IGF-1Ea or IGF-1Eb expression in muscle, activating a series of anabolic and compensatory pathways, is able to avoid muscle loss and a normal muscle-nerve interaction [47]. IGFBP belongs to a family of soluble proteins having a high affinity to bind with IGF-1 and 2. In humans, IGFBP 3 is the most abundant IGFBP and binds with a maximum amount of circulating IGF-1 [28]. The half-life from minutes to ∼15 h is extended upon the incorporation of IGF-1 into the ternary complex, thus creating a stable pool of IGF-1 inside the circulation; which, further combined with the other IGFBP, can provide subtle regulation of the availability of IGF-1 to target tissues [48,49].

## 2. Role of IGF-1 in Skeletal Muscle

IGF-1 plays a critical role in myogenesis during embryonic development, although the mechanism responsible for IGF-1 mediated myoblast proliferation remains unclear [50]. Aging, ischemia, cancer, motor neuron degeneration, and heart failure are all associated with SM loss, for which there is no effective treatment. IGF-1 production plays an important role in muscle healing and maintenance. Preclinical experiments have shown that IGF-1 is associated with muscle mass and strength development, it reduces muscle degeneration, prevents excessive toxin-induced inflammatory expansion, and increases the proliferation capacity of muscle satellite cells (MSCs) [35]. MSCs are key players in SM regeneration [12], and IGF-1 is also produced in SM to control muscle growth in a paracrine/autocrine manner [51]. IGF-1 is also a biomarker of health and fitness; in fact, higher circulating IGF-1 concentrations are positively related to health factors associated with body structure and cardiovascular strength, and negatively related to body fat levels. Aerobic fitness and muscular stamina are positively associated with circulating IGF-1 concentrations [52]. Malnutrition, sepsis, critical sickness, high doses of exogenous glucocorticoids and inflammation, are responsible to lower the IGF-1 mRNA in muscle [51]. Like IGF-1, IGF-2 is also essential for muscle differentiation and development and acts in an autocrine manner [53]. Transforming growth factor-beta1 (TGF-β1) has been reported to diminish IGF-2 gene expression in myoblasts, decrease IGF-2 secretion, and reduce IGF-1 receptor activation [54].

## 3. Mechanism of IGF-1 in Skeletal Muscle

**Figure 2:**
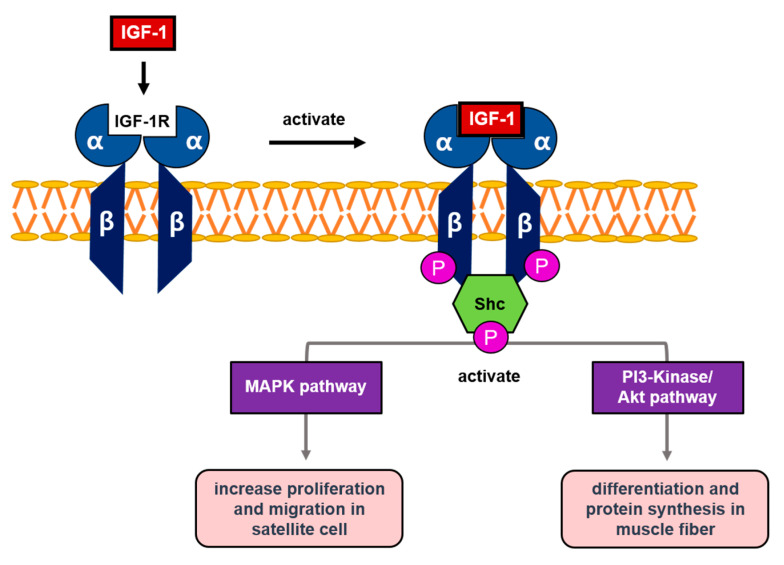
The molecular mechanism of IGF-1. IGF-1 interacts with its receptor (IGF-1R), and thus, activates the PI3K/Akt and mitogen-activated protein kinase (MAPK) pathways, which regulate MSC proliferation and differentiation.

Several tissues secrete IGF-1, and the actions of IGF-1 appear to be dependent on the secretory site. Most IGF-1, also known as “somatomedin C”, is secreted by the liver and transported as an endocrine hormone to other tissues [55]. The IGF-1 cascade is mediated by its interaction with IGF-1R, which has transmembrane locations and tyrosine kinase-like activity [51]. IGF-1R acts as a phosphatidylinositol 3-kinase/protein kinase B (PI3K/Akt) pathway activator and its expression is associated with myoblast proliferation and normal muscle mass maintenance [56] (Figure 2).

It has been reported that the mitogenic activity of IGF-1 on myoblast cells is crucial and mediated by two main signaling pathways, that is, the mitogen-activated protein kinase (MAPK/ERK1/2) pathway and the PI3K/Akt pathway, which are both associated with cell cycle progression and cell survival [57]. Furthermore, the Akt-facilitated growth effect of IGF-1 in SM appears to promote protein synthesis and muscle cell development [58,59]. The PI3K-Akt cascade is the main IGF-1 signal activated in muscle. Akt1/Akt2 double-knockout mice and IGF-1R knockout mice displayed a severe growth deficiency. They both exhibited a decreased SM mass, although IGF-1R knockout mice attributed to a decrease in the number of muscle cells, whereas in the Akt1/Akt2 double-knockout mice attributed mostly to a decrease in individual cell size and suggested that IGF-1R functions during development are mostly dependent on Akt [60]. IGF-1 plays an essential role in myoblast proliferation and differentiation, and protects cells from apoptosis [61]. In the heterotetramer structure of IGF-1R, two subunits are responsible for IGF-1 binding and the other two subunits exhibit tyrosine kinase-like activity. The IGF-1 binding capability of the ligand-binding area of IGF-1R has a six-fold greater attraction for IGF-1 than IGF-2. After binding IGF-1, the intrinsic tyrosine kinase of IGF-1R autophosphorylates tyrosines that then act as docking positions for signaling proteins, which include insulin receptor substrate-1 (IRS-1). IGF-1R also phosphorylates Shc, which subsequently triggers the RAS/MAP kinase pathway to prompt mitogenesis. Muscle injury enhances IGF-1 synthesis by MSCs in rodents, which stimulates MSC proliferation and differentiation to myoblasts [35,62,63]. Mechanical loading also affects the production of IGF-1 by SM [51,64].

## 4. Relationship Between IGF-1 and Myostatin

IGFs and myostatin (MSTN) have contrasting roles in the regulation of SM size and growth, in particular, MSTN inhibits SM growth [65]. Circulating MSTN-attenuating mediators are being developed to treat muscle-wasting ailments, as MSTN/activin receptors are widely distributed among many nonmuscle tissues [66]. Follistatin is an inhibitor of MSTN and induces dramatic SM mass increases, upon the IGF-1 receptor/Akt/mTOR cascade [67].

IGF-1 knockout mice show muscle hypoplasia [68]. Moreover, inhibition of MSTN stimulates the Akt/mTOR/S6K pathway, which is essential for the muscle hypertrophy initiated by IGF-1 [69,70,71]. The regulation of IGF-1 during the muscle hypertrophy induced by MSTN inhibition is still disputed. Elevated expressions of muscle mRNA and circulating concentrations of IGF-1 were observed following MSTN inhibition [67]. Morissette et al. reported that Akt protein levels were high in SMs of MSTN knockout mice [70].

## 5. Role of IGF-1 in Different Diseases

### 5.1. Role of IGF-1 in Duchenne Muscular Dystrophy

Duchenne muscular dystrophy (DMD) is a form of muscular dystrophy associated with X-linked recessive disorder caused by mutation of dystrophin in SM [72,73]. DMD shows a male predominance and causes muscle degeneration. Several studies have demonstrated extremely encouraging outcomes for IGF-1 treatment in DMD [74,75]. Moreover, muscle and circulating levels of IGF-1 frequently reduce in response to glucocorticoids [76]. In vitro study by Fang et al. demonstrated that glucocorticoid and IGF-1 cotreatment participate in myogenic differentiation through the Akt/GSK-3β pathway in C2C12 myoblasts. It revealed that increased phosphorylated Ser473-Akt and phosphorylated Ser9-GSK-3b as well as myogenic differentiation, provide a way for a potential alternative strategy to DMD treatment [76]. IGF-1 has been recommended for patients experiencing muscle-wasting conditions [77] and various studies have explored the functional properties of dystrophic SM after IGF-1 treatment. Lynch et al. found that four weeks of IGF-1 treatment (~2 mg/kg body mass, 50 g/h delivered subcutaneously by a miniosmotic pump) increased the mass and force-producing limit of SM from dystrophic mice. Furthermore, IGF-1 increased extensor digitorum longus (EDL) and soleus muscle masses of dystrophic mice by 20% and 29%, respectively, as compared with untreated dystrophic controls [77].

### 5.2. Role of IGF-1 in Muscle Atrophy

Muscle atrophy (MA) is defined as a loss of muscle mass and quality, and it is encountered in several disease conditions, for example, in malignancies, AIDS, congestive cardiovascular breakdown, chronic obstructive pulmonary disease, and renal failure and in serious burn patients [78]. Anabolic-androgenic steroids and different hormones, such as GH and IGF-1 appear to increase muscle mass in patients with MA [79]. Lama2-linked muscular dystrophy is a serious congenital muscular dystrophy produced by mutations in the LAMA2 gene, and is associated with several pathological problems such as inflammation, apoptosis, fibrosis, necrosis, severe muscle weakness, and subnominal postnatal growth. As indicated by Accorsi et al. losartan combinatorial management appeared to enhance transgenic IGF-1 overexpression, recover postnatal growth, reduce inflammation and fibrosis, increase body weights, and result in a remarkable restoration of muscle architecture and locomotory ability in DyW mice (mouse model of Lama2-related muscular dystrophy) [80].

### 5.3. Role of IGF-1 in Cancer

Increases in IGF-1R activity promote cancer cell proliferation, migration, and invasion and are related to tumor metastasis, treatment resistance, and reduced survival [81]. IGFBP2 has been identified as a prominent oncogene in most epithelial cancers [82]. A number of authors have proposed IGFBP2 viewed as a potential target for regulating cancer metastasis and invasion-related signaling networks, though its mode of action is keenly debated [83]. IGF-1 has been reported to upregulate angiogenesis and tumor invasion by activating matrix metalloproteinases [84], which are well known nonglycolytic proteolytic enzyme biomarkers in several cancer types [85]. Currently, therapies targeting the IGF system have attracted considerable attention in cancer research. The proliferative, antiapoptotic, and transformative impacts of IGFs are primarily activated by IGF-1R ligation [86]. Higher levels of serum IGF-1 are linked with increased risk of several common cancers comprising breast, colorectal, and prostate [87].

### 5.4. Role of IGF-1 in Neurodegeneration

Neurodegenerative diseases like Alzheimer’s, Parkinson’s diseases and prion disorders are associated with aging [88,89,90,91,92]. A number of promising results show that IGF-1 has a restorative impact on the brain by expanding hippocampal neurogenesis and memory accuracy in older people and potentially in individuals with neurodegenerative disorders [93]. IGF-1 has a progressively more powerful trophic impact than GH on sensory and motor neurons and on neuronal growth and recovery. IGF-1 stimulates neurite development and assumes an essential role during central and peripheral nervous system development [94,95].

It can be summarized that IGF-1 plays a crucial role in the management of various diseases and could be used in the therapeutic possibilities of several diseases, including DMD, muscle atrophy, etc. Recent IGF-1 studies are detailed in Table 1, which clearly showed the role of IGF-1 in various areas such as SM regeneration, tissue recovery, depression pathophysiology, etc.

## 6. Interaction Between IGF-1 and IGF-1R

**Figure 3:**
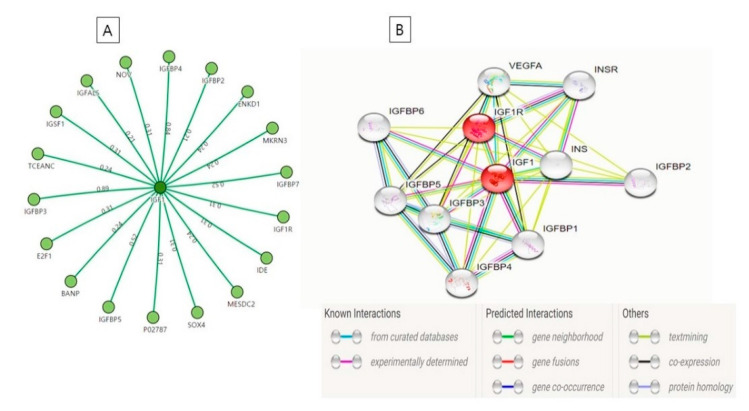
Protein-Protein interactions of IGF-1 with its associated proteins generated by (**A**) SIGnaling Network Open Resource, (**B**) STRING.

**Figure 4:**
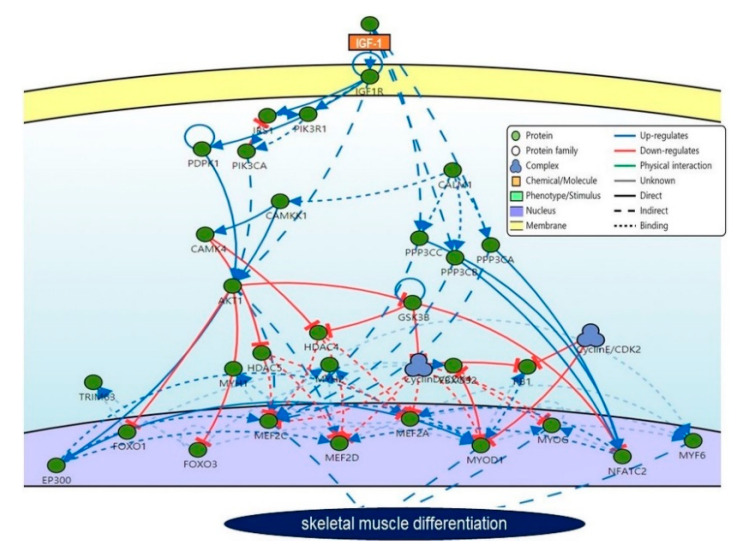
The mechanistic role of IGF-1 during skeletal muscle differentiation. The figure shows signaling interactions during muscle differentiation as predicted by SIGnaling Network Open Resource (SIGNOR).

Protein-protein interactions (PPIs) provide graphical illustrations of interactions between two or more proteins. PPI strategy plays an important role in the body for metabolic and signaling processes. A better understanding of the interaction between IGF-1 and IGF-1R along with several other associated proteins (Figure 3A) was obtained by SIGnaling Network Open Resource (SIGNOR; http://signor.uniroma2.it). The SIGNOR web tool can be used to predict activation/inactivation, interactions, and connections between biomolecules and signaling molecules [104]. GFs and other membrane-bound entities (e.g., ECM molecules) activate transmembrane receptors that trigger signaling responses that eventually regulate gene expressions and metabolic processes (Figure 4).

The STRING database (http://string-db.org) enables critical assessments or direct (physical) and indirect (functional) PPIs. By using STRING [105], we were able to identify interacting nodes between IGF-1 and IGF-1R (Figure 3B). The interactions generated by the STRING are based on the known interactions (from the curated databases and experimentally determined), predicted interactions (e.g., gene neighborhood and gene co-occurrence) as well as few other factors viz. text mining, coexpression, etc. In this interaction, several other associated proteins such as IGFBP 1 to 6, insulin (INS), insulin to its receptor (INSR), and vascular endothelial growth factor A (VEGFA) were found to interact with each other through IGF-1 and IGF1R. Black lines represent the coexpression while the light blue line represents the protein homology. Text-mining data represents the association between proteins as shown in Figure 3B. The half-life of the IGFs are prolonged by IGFBP and helped in the growth-promoting effects of the IGFs on cell culture. INS decreases blood glucose and increases cell permeability to amino acids, monosaccharides, and fatty acids. Binding of insulin to its receptor (INSR) leads to phosphorylation of intracellular substrates, such as insulin receptor substrates (IRS1, 2, 3, 4), SHC, GAB1, and other signaling intermediates. Each of these phosphorylated proteins serve as docking proteins for other signaling proteins. VEGFA is active in angiogenesis, vasculogenesis and endothelial cell growth, it induces endothelial cell proliferation, promotes cell migration, inhibits apoptosis and induces permeabilization of blood vessels (http://string-db.org).

In Figure 4, the green circle represents the protein which binds with its receptor to direct the signaling path inside the cell. IGF-1 interaction is clearly shown in different parts of cells such as membrane to the nucleus. In this figure, the red line represents the downregulation while upregulation is represented by the blue line. The dotted line represents the binding mode between the intermediates.

**Figure 5:**
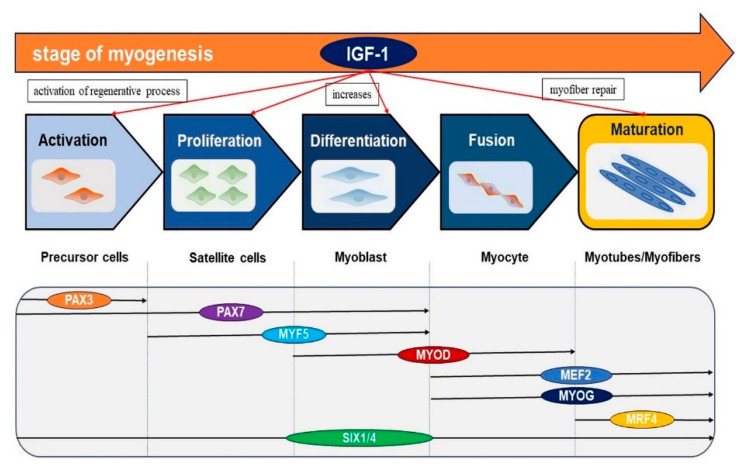
Role of IGF-1 in myogenesis. IGF-1 is activated during muscle regeneration and increases MSC proliferation and differentiation. In addition, IGF-1 promotes myofiber repairs.

The functions of proteins associated with IGF-1 are provided in Table 2, and the role played by IGF-1 in myogenesis is depicted schematically in Figure 5. The different myogenic regulatory factors such as Pax3, Pax7, MyoD, Myf5, MyoG, and Mrf4 genes are collectively expressed in the SM lineage in different tissues during development [106,107]. IGF-1 plays an important role in the activation of precursor cells and helps in the activation of the regenerative process. IGF-1 also increases the proliferation and differentiation of satellite cells and myoblast respectively. IGF-1 helps in myofiber repair. In precise, IGF-1 can favor regenerative myogenesis and support the robustness of myofibers [108]. Collectively, IGF-1 is helpful in satellite cell proliferation and differentiation. Skeletal myogenesis is an extraordinarily complex process, which is regulated at multiple levels, and transcriptional regulation naturally plays an important role during muscle formation.

The structure obtained by the SIGNOR network (Figure 3A) is showing the different proteins which are interlinked to IGF-1. These proteins are listed in the left part of Table 2. Now, here authors tried to elaborate in a single word about the function of these proteins as mentioned in the right part of Table 2. The IGFBP family consists of six IGFBPs, namely IGFBP1 to IGFBP6, however other proteins with low binding affinity to IGFs were known as IGFBP7, IGFBP8, IGFBP9 [109].

Overall GH is known to stimulate growth in children and adolescents with various metabolic functions [112]. Musculoskeletal injuries represent a major public health problem [113], and medications improve muscle repair and restore functions. Increasing IGF-1 levels improves SM recovery after myotoxic injury and the administration of IGF-1 has the potential for accelerating healing after trauma [114].

## 7. Concluding Remarks

IGF-1 plays an important role in the maintenance of muscle mass by acting in paracrine, autocrine, or endocrine manners. GH upregulates IGF-1 synthesis in the liver, and thereby, increases its plasma concentrations. IGF-1 is the main stimulator of SM mass since this hormone increases protein synthesis and decreases proteolysis. In addition, IGF-1 increases MSC proliferation and myoblast proliferation and differentiation during normal growth or regeneration after SM injury. Therefore, IGF-1 increases SM mass and muscle functional capacities. In addition, IGF-1 plays an important role in the prevention of muscle atrophy. The development of IGF-1 for the treatment of muscle-wasting conditions remains an important research challenge.

## Figures and Tables

**Table 1:** Recent research studies on IGF-1 in different fields.

S. No.	Role of IGF-1	Year	References
1.	IGF-1 helps in the growth and regeneration of SM and bones. Its signaling in the smooth muscle cell and in fibroblast is a critical factor of normal vascular wall growth and atheroprotection.	2020	[96,97]
2.	IGF-1 helps in the activation of IGF-1R and muscle tissue recovery. Shapiro et al. indicate that the IGFBP-3/IGF1 conjugated framework has the potential to be utilized for in-situ muscle tissue recovery.	2019	[98,99]
3.	IGF-1 have pleiotropic consequences on the skeleton during the life expectancy by prompting the bone development and resorption. Lower IGF-1 levels are related to lower handgrip strength and more terrible physical execution.	2018	[100,101]
4.	GH/IGF-1 treatment had various impacts on patients with traumatic brain injury, proving a high recuperation of neurons and clinical results.	2017	[95]
5.	IGF-1 appear in the regulation of neuronal harm, toxic insults, and a few other neurodegenerative procedures.	2016	[102]
6.	According to Kopczak et al., the signaling of IGF-1 could play a role in the pathophysiology of depression.	2015	[103]

**Table 2:** Function of IGF-1 related proteins.

S. No.	Name	Function
1.	IGF-1R	Cell growth and survival control
2.	IGFBP3 and IGFBP4	Enhance the capability of IGF-1 to promote cell growth
3.	Probable E3 ubiquitin-protein ligase makorin-3 (MKRN3)	Catalyze the covalent interactions of ubiquitin moieties onto substrate proteins
4.	IGFBP-complex acid-labile subunit (IGFALS)	Regulation of the circulation of IGFs and receptor-ligand binding [110]
5.	Protein BANP	Cell cycle arrest
6.	Transcription factor E2F1	Mediate cell proliferation
7	Transcription factor SOX-4	High-affinity binding to the T-cell enhancer motif 5’-AACAAAG-3’ motif
8.	IGFBP7	Stimulates cell adhesion
9.	IGFBP5	Change the interaction of IGFs with their cell surface receptors.
10.	Immunoglobulin superfamily member 1 (IGSF1)	Essential to mediate a specific antagonistic effect of inhibin B on activin-stimulated transcription
11.	Insulin-degrading enzyme	Cellular breakdown of insulin
12.	IGFBP1, IGFBP3, IGFBP5	Stimulate IGF actions
13.	LDLR chaperone MESD (low-density lipoprotein receptors)	Help in embryonic polarity and mesoderm induction
14.	Protein NOV homolog (IGFBP9)	Binds with integrins or other membrane receptors e.g., NOTCH1 [111]

## Abbreviations

SM	skeletal muscle
GFs	growth factors
ECM	extracellular matrix
MSC	muscle satellite cell
IGF-1	insulin-like growth factor-1
IGF-1R	insulin-like growth factor type 1 receptor
TGF-β	transforming growth factor beta
GH	growth hormone
MSTN	myostatin
DMD	Duchenne muscular dystrophy
MA	muscle atrophy
IGFBP	IGF binding proteins

## References

[1] Frontera W.R., Ochala J. (2015). Skeletal muscle: A brief review of structure and function. Calcif. Tissue Int..

[2] Kim T., Ahmad K., Shaikh S., Jan A.T., Seo M.G., Lee E.J., Choi I. (2019). Dermatopontin in skeletal muscle extracellular matrix regulates myogenesis. Cells.

[3] Langlois S., Cowan K.N. (2017). Regulation of skeletal muscle myoblast differentiation and proliferation by pannexins. Adv. Exp. Med. Biol..

[4] Lee E.J., Pokharel S., Jan A.T., Huh S., Galope R., Lim J.H., Lee D.M., Choi S.W., Nahm S.S., Kim Y.W. (2017). Transthyretin: A transporter protein essential for proliferation of myoblast in the myogenic program. Int. J. Mol. Sci..

[5] Halper J. (2010). Growth factors as active participants in carcinogenesis: A perspective. Vet. Pathol..

[6] Vlasova-St Louis I., Bohjanen P.R. (2017). Post-transcriptional regulation of cytokine and growth factor signaling in cancer. Cytokine Growth Factor Rev..

[7] Chen F.M., Zhang M., Wu Z.F. (2010). Toward delivery of multiple growth factors in tissue engineering. Biomaterials.

[8] Macri L., Clark R.A. (2009). Tissue engineering for cutaneous wounds: Selecting the proper time and space for growth factors, cells and the extracellular matrix. Skin Pharmacol. Physiol..

[9] Han G., Ceilley R. (2017). Chronic wound healing: A review of current management and treatments. Adv. Ther..

[10] Lee E.J., Jan A.T., Baig M.H., Ashraf J.M., Nahm S.-S., Kim Y.-W., Park S.-Y., Choi I. (2016). Fibromodulin: A master regulator of myostatin controlling progression of satellite cells through a myogenic program. FASEB J..

[11] Lee E.J., Nam J.H., Choi I. (2018). Fibromodulin modulates myoblast differentiation by controlling calcium channel. Biochem. Biophys. Res. Commun..

[12] Lee E.J., Jan A.T., Baig M.H., Ahmad K., Malik A., Rabbani G., Kim T., Lee I.K., Lee Y.H., Park S.Y. (2018). Fibromodulin and regulation of the intricate balance between myoblast differentiation to myocytes or adipocyte-like cells. FASEB J..

[13] Ahmad S., Jan A.T., Baig M.H., Lee E.J., Choi I. (2017). Matrix gla protein: An extracellular matrix protein regulates myostatin expression in the muscle developmental program. Life Sci..

[14] Schreml S., Szeimies R.M., Prantl L., Landthaler M., Babilas P. (2010). Wound healing in the 21st century. J. Am. Acad. Dermatol..

[15] Posnett J., Gottrup F., Lundgren H., Saal G. (2009). The resource impact of wounds on health-care providers in Europe. J. Wound Care.

[16] Menke N.B., Ward K.R., Witten T.M., Bonchev D.G., Diegelmann R.F. (2007). Impaired wound healing. Clin. Dermatol..

[17] Robson M.C., Mustoe T.A., Hunt T.K. (1998). The future of recombinant growth factors in wound healing. Am. J. Surg..

[18] Park J.W., Hwang S.R., Yoon I.S. (2017). Advanced growth factor delivery systems in wound management and skin regeneration. Molecules.

[19] Werner S., Grose R. (2003). Regulation of wound healing by growth factors and cytokines. Physiol. Rev..

[20] Kim S.H., Kiick K.L. (2007). Heparin-mimetic sulfated peptides with modulated affinities for heparin-binding peptides and growth factors. Peptides.

[21] Nimni M.E. (1997). Polypeptide growth factors: Targeted delivery systems. Biomaterials.

[22] Torchilin V.P., Lukyanov A.N. (2003). Peptide and protein drug delivery to and into tumors: Challenges and solutions. Drug Discov. Today.

[23] Edelman E.R., Nugent M.A., Smith L.T., Karnovsky M.J. (1992). Basic fibroblast growth factor enhances the coupling of intimal hyperplasia and proliferation of vasa vasorum in injured rat arteries. J. Clin. Invest..

[24] Gospodarowicz D., Cheng J. (1986). Heparin protects basic and acidic FGF from inactivation. J. Cell. Physiol..

[25] Roghani M., Mansukhani A., Dell’Era P., Bellosta P., Basilico C., Rifkin D.B., Moscatelli D. (1994). Heparin increases the affinity of basic fibroblast growth factor for its receptor but is not required for binding. J. Biol. Chem..

[26] Liao I.C., Wan A.C., Yim E.K., Leong K.W. (2005). Controlled release from fibers of polyelectrolyte complexes. J. Control. Release.

[27] Lee A.C., Yu V.M., Lowe J.B., Brenner M.J., Hunter D.A., Mackinnon S.E., Sakiyama-Elbert S.E. (2003). Controlled release of nerve growth factor enhances sciatic nerve regeneration. Exp. Neurol..

[28] Martin A.I., Priego T., Lopez-Calderon A. (2018). Hormones and muscle atrophy. Adv. Exp. Med. Biol..

[29] Le Roith D. (1997). Seminars in medicine of the Beth Israel Deaconess Medical Center. Insulin-like growth factors. N. Engl. J. Med..

[30] Zhang X., Yee D. (2000). Tyrosine kinase signalling in breast cancer: Insulin-like growth factors and their receptors in breast cancer. Breast Cancer Res..

[31] Daughaday W.H., Rotwein P. (1989). Insulin-like growth factors I and II. Peptide, messenger ribonucleic acid and gene structures, serum, and tissue concentrations. Endocr. Rev..

[32] Baker J., Liu J.P., Robertson E.J., Efstratiadis A. (1993). Role of insulin-like growth factors in embryonic and postnatal growth. Cell.

[33] Sell C., Dumenil G., Deveaud C., Miura M., Coppola D., DeAngelis T., Rubin R., Efstratiadis A., Baserga R. (1994). Effect of a null mutation of the insulin-like growth factor I receptor gene on growth and transformation of mouse embryo fibroblasts. Mol. Cell. Biol..

[34] Vitale G., Pellegrino G., Vollery M., Hofland L.J. (2019). ROLE of IGF-1 system in the modulation of longevity: Controversies and new insights from a centenarians’ perspective. Front. Endocrinol..

[35] Song Y.H., Song J.L., Delafontaine P., Godard M.P. (2013). The therapeutic potential of IGF-I in skeletal muscle repair. Trends Endocrinol. Metab..

[36] Ikemoto-Uezumi M., Uezumi A., Tsuchida K., Fukada S., Yamamoto H., Yamamoto N., Shiomi K., Hashimoto N. (2015). Pro-insulin-like growth factor-ii ameliorates age-related inefficient regenerative response by orchestrating self-reinforcement mechanism of muscle regeneration. Stem Cells.

[37] Annibalini G., Bielli P., De Santi M., Agostini D., Guescini M., Sisti D., Contarelli S., Brandi G., Villarini A., Stocchi V. (2016). MIR retroposon exonization promotes evolutionary variability and generates species-specific expression of IGF-1 splice variants. Biochim. Biophys. Acta.

[38] Matheny R.W., Nindl B.C. (2011). Loss of IGF-IEa or IGF-IEb impairs myogenic differentiation. Endocrinology.

[39] Cheema U., Brown R., Mudera V., Yang S.Y., McGrouther G., Goldspink G. (2005). Mechanical signals and IGF-I gene splicing in vitro in relation to development of skeletal muscle. J. Cell. Physiol..

[40] Sandona D., Desaphy J.F., Camerino G.M., Bianchini E., Ciciliot S., Danieli-Betto D., Dobrowolny G., Furlan S., Germinario E., Goto K. (2012). Adaptation of mouse skeletal muscle to long-term microgravity in the MDS mission. PLoS ONE.

[41] Kineman R.D., Del Rio-Moreno M., Sarmento-Cabral A. (2018). 40 YEARS of IGF1: Understanding the tissue-specific roles of IGF1/IGF1R in regulating metabolism using the Cre/loxP system. J. Mol. Endocrinol..

[42] De Santi M., Annibalini G., Barbieri E., Villarini A., Vallorani L., Contarelli S., Berrino F., Stocchi V., Brandi G. (2016). Human IGF1 pro-forms induce breast cancer cell proliferation via the IGF1 receptor. Cell. Oncol..

[43] Ohlsson C., Mohan S., Sjogren K., Tivesten A., Isgaard J., Isaksson O., Jansson J.O., Svensson J. (2009). The role of liver-derived insulin-like growth factor-I. Endocr. Rev..

[44] Wan X., Wang S., Xu J., Zhuang L., Xing K., Zhang M., Zhu X., Wang L., Gao P., Xi Q. (2017). Dietary protein-induced hepatic IGF-1 secretion mediated by PPARgamma activation. PLoS ONE.

[45] Savage M.O. (2013). Insulin-like growth factors, nutrition and growth. World Rev. Nutr. Diet..

[46] Kjobsted R., Hingst J.R., Fentz J., Foretz M., Sanz M.N., Pehmoller C., Shum M., Marette A., Mounier R., Treebak J.T. (2018). AMPK in skeletal muscle function and metabolism. FASEB J..

[47] Ascenzi F., Barberi L., Dobrowolny G., Villa Nova Bacurau A., Nicoletti C., Rizzuto E., Rosenthal N., Scicchitano B.M., Musaro A. (2019). Effects of IGF-1 isoforms on muscle growth and sarcopenia. Aging Cell.

[48] Firth S.M., Baxter R.C. (2002). Cellular actions of the insulin-like growth factor binding proteins. Endocr. Rev..

[49] Williams R.M., McDonald A., O’Savage M., Dunger D.B. (2008). Mecasermin rinfabate: rhIGF-I/rhIGFBP-3 complex: iPLEX. Expert Opin. Drug. Metab. Toxicol..

[50] Yu M., Wang H., Xu Y., Yu D., Li D., Liu X., Du W. (2015). Insulin-like growth factor-1 (IGF-1) promotes myoblast proliferation and skeletal muscle growth of embryonic chickens via the PI3K/Akt signalling pathway. Cell Biol. Int..

[51] Clemmons D.R. (2009). Role of IGF-I in skeletal muscle mass maintenance. Trends Endocrino.l Metab..

[52] Nindl B.C., Santtila M., Vaara J., Hakkinen K., Kyrolainen H. (2011). Circulating IGF-I is associated with fitness and health outcomes in a population of 846 young healthy men. Growth Horm. IGF Res..

[53] Alzhanov D.T., McInerney S.F., Rotwein P. (2010). Long range interactions regulate Igf2 gene transcription during skeletal muscle differentiation. J. Biol. Chem..

[54] Gardner S., Alzhanov D., Knollman P., Kuninger D., Rotwein P. (2011). TGF-beta inhibits muscle differentiation by blocking autocrine signaling pathways initiated by IGF-II. Mol. Endocrinol..

[55] Laron Z. (2001). Insulin-like growth factor 1 (IGF-1): A growth hormone. Mol. Pathol..

[56] Hamilton D.L., Philp A., MacKenzie M.G., Baar K. (2010). A limited role for PI(3,4,5)P3 regulation in controlling skeletal muscle mass in response to resistance exercise. PLoS ONE.

[57] Fu S., Yin L., Lin X., Lu J., Wang X. (2018). Effects of cyclic mechanical stretch on the proliferation of L6 myoblasts and its mechanisms: PI3K/Akt and MAPK signal pathways regulated by IGF-1 receptor. Int. J. Mol. Sci..

[58] Sandri M., Barberi L., Bijlsma A.Y., Blaauw B., Dyar K.A., Milan G., Mammucari C., Meskers C.G., Pallafacchina G., Paoli A. (2013). Signalling pathways regulating muscle mass in ageing skeletal muscle: The role of the IGF1-Akt-mTOR-FoxO pathway. Biogerontology.

[59] Liu M., Zhang S. (2011). Amphioxus IGF-like peptide induces mouse muscle cell development via binding to IGF receptors and activating MAPK and PI3K/Akt signaling pathways. Mol. Cell. Endocrinol..

[60] Peng X.D., Xu P.Z., Chen M.L., Hahn-Windgassen A., Skeen J., Jacobs J., Sundararajan D., Chen W.S., Crawford S.E., Coleman K.G. (2003). Dwarfism, impaired skin development, skeletal muscle atrophy, delayed bone development, and impeded adipogenesis in mice lacking Akt1 and Akt2. Genes Dev..

[61] Borselli C., Storrie H., Benesch-Lee F., Shvartsman D., Cezar C., Lichtman J.W., Vandenburgh H.H., Mooney D.J. (2010). Functional muscle regeneration with combined delivery of angiogenesis and myogenesis factors. Proc. Natl. Acad. Sci. USA.

[62] Barton-Davis E.R., Shoturma D.I., Sweeney H.L. (1999). Contribution of satellite cells to IGF-I induced hypertrophy of skeletal muscle. Acta Physiol. Scand..

[63] Edwall D., Schalling M., Jennische E., Norstedt G. (1989). Induction of insulin-like growth factor I messenger ribonucleic acid during regeneration of rat skeletal muscle. Endocrinology.

[64] Yang S., Alnaqeeb M., Simpson H., Goldspink G. (1996). Cloning and characterization of an IGF-1 isoform expressed in skeletal muscle subjected to stretch. J. Muscle Res. Cell Motil..

[65] Hennebry A., Oldham J., Shavlakadze T., Grounds M.D., Sheard P., Fiorotto M.L., Falconer S., Smith H.K., Berry C., Jeanplong F. (2017). IGF1 stimulates greater muscle hypertrophy in the absence of myostatin in male mice. J. Endocrinol..

[66] Czaja W., Nakamura Y.K., Li N., Eldridge J.A., DeAvila D.M., Thompson T.B., Rodgers B.D. (2019). Myostatin regulates pituitary development and hepatic IGF1. Am. J. Physiol. Endocrinol. Metab..

[67] Barbe C., Kalista S., Loumaye A., Ritvos O., Lause P., Ferracin B., Thissen J.P. (2015). Role of IGF-I in follistatin-induced skeletal muscle hypertrophy. Am. J. Physiol. Endocrinol. Metab..

[68] Liu J.P., Baker J., Perkins A.S., Robertson E.J., Efstratiadis A. (1993). Mice carrying null mutations of the genes encoding insulin-like growth factor I (Igf-1) and type 1 IGF receptor (Igf1r). Cell.

[69] Lipina C., Kendall H., McPherron A.C., Taylor P.M., Hundal H.S. (2010). Mechanisms involved in the enhancement of mammalian target of rapamycin signalling and hypertrophy in skeletal muscle of myostatin-deficient mice. FEBS Lett..

[70] Morissette M.R., Cook S.A., Buranasombati C., Rosenberg M.A., Rosenzweig A. (2009). Myostatin inhibits IGF-I-induced myotube hypertrophy through Akt. Am. J. Physiol. Cell Physiol..

[71] Rommel C., Bodine S.C., Clarke B.A., Rossman R., Nunez L., Stitt T.N., Yancopoulos G.D., Glass D.J. (2001). Mediation of IGF-1-induced skeletal myotube hypertrophy by PI(3)K/Akt/mTOR and PI(3)K/Akt/GSK3 pathways. Nat. Cell Biol..

[72] Suthar R., Sankhyan N. (2018). Duchenne muscular dystrophy: A practice update. Indian J. Pediatr..

[73] Ahmad K., Shaikh S., Ahmad S.S., Lee E.J., Choi I. (2020). Cross-talk between extracellular matrix and skeletal muscle: Implications for myopathies. Front. Pharmacol..

[74] Noguchi S. (2005). The biological function of insulin-like growth factor-I in myogenesis and its therapeutic effect on muscular dystrophy. Acta Myol..

[75] Patel K., Macharia R., Amthor H. (2005). Molecular mechanisms involving IGF-1 and myostatin to induce muscle hypertrophy as a therapeutic strategy for Duchenne muscular dystrophy. Acta Myol..

[76] Fang X.B., Song Z.B., Xie M.S., Liu Y.M., Zhang W.X. (2020). Synergistic effect of glucocorticoids and IGF-1 on myogenic differentiation through the Akt/GSK-3beta pathway in C2C12 myoblasts. Int. J. Neurosci..

[77] Lynch G.S., Cuffe S.A., Plant D.R., Gregorevic P. (2001). IGF-I treatment improves the functional properties of fast- and slow-twitch skeletal muscles from dystrophic mice. Neuromuscul. Disord..

[78] Cao R.Y., Li J., Dai Q., Li Q., Yang J. (2018). Muscle Atrophy: Present and Future. Adv. Exp. Med. Biol..

[79] Fink J., Schoenfeld B.J., Nakazato K. (2018). The role of hormones in muscle hypertrophy. Phys. Sportsmed..

[80] Accorsi A., Kumar A., Rhee Y., Miller A., Girgenrath M. (2016). IGF-1/GH axis enhances losartan treatment in Lama2-related muscular dystrophy. Hum. Mol. Genet..

[81] Sun Y., Sun X., Shen B. (2017). Molecular Imaging of IGF-1R in Cancer. Mol. Imaging.

[82] Pickard A., McCance D.J. (2015). IGF-Binding Protein 2—Oncogene or Tumor Suppressor?. Front. Endocrinol..

[83] Yao X., Sun S., Zhou X., Guo W., Zhang L. (2016). IGF-binding protein 2 is a candidate target of therapeutic potential in cancer. Tumour Biol..

[84] Thant A.A., Wu Y., Vadgama J.V. (2005). Cancer invasion and angiogenesis by IGF-1-induced MMP-2 and VEGF in human Breast cancer cells. Proc. Am. Assoc. Cancer Res..

[85] Baig M.H., Adil M., Khan R., Dhadi S., Ahmad K., Rabbani G., Bashir T., Imran M.A., Husain F.M., Lee E.J. (2019). Enzyme targeting strategies for prevention and treatment of cancer: Implications for cancer therapy. Semin. Cancer Biol..

[86] Philippou A., Christopoulos P.F., Koutsilieris D.M. (2017). Clinical studies in humans targeting the various components of the IGF system show lack of efficacy in the treatment of cancer. Mutat. Res. Rev. Mutat. Res..

[87] Major J.M., Laughlin G.A., Kritz-Silverstein D., Wingard D.L., Barrett-Connor E. (2010). Insulin-like growth factor-I and cancer mortality in older men. J. Clin. Endocrinol. Metab..

[88] Ahmad S.S., Khan H., Danish Rizvi S.M., Ansari S.A., Ullah R., Rastrelli L., Mahmood H.M., Siddiqui M.H. (2019). Computational study of natural compounds for the clearance of amyloid-betaeta: A potential therapeutic management strategy for alzheimer’s disease. Molecules.

[89] Ahmad S.S., Kamal M.A. (2019). Current updates on the regulation of beta-secretase movement as a potential restorative focus for management of alzheimer’s disease. Protein Pept. Lett..

[90] Waldthaler J., Timmermann L. (2019). Update on diagnostics and therapy of idiopathic Parkinson’s disease. Fortschr. Neurol. Psychiatr..

[91] Ahmad S.S., Waheed T., Rozeen S., Mahmood S., Kamal M.A. (2019). Therapeutic study of phytochemicals against cancer and alzheimer’s disease management. Curr. Drug Metab..

[92] Walker L.C., Jucker M. (2015). Neurodegenerative diseases: Expanding the prion concept. Annu. Rev. Neurosci..

[93] Morel G.R., Leon M.L., Uriarte M., Reggiani P.C., Goya R.G. (2017). Therapeutic potential of IGF-I on hippocampal neurogenesis and function during aging. Neurogenesis.

[94] Rosenbloom A.L., Rivkees S.A. (2010). Off-label use of recombinant igf-I to promote growth: Is it appropriate?. J. Clin. Endocrinol. Metab..

[95] Bianchi V.E., Locatelli V., Rizzi L. (2017). Neurotrophic and neuroregenerative effects of GH/IGF1. Int. J. Mol. Sci..

[96] Park J., Yan G., Kwon K.C., Liu M., Gonnella P.A., Yang S., Daniell H. (2020). Oral delivery of novel human IGF-1 bioencapsulated in lettuce cells promotes musculoskeletal cell proliferation, differentiation and diabetic fracture healing. Biomaterials.

[97] Sukhanov S., Higashi Y., Shai S.Y., Snarski P., Danchuk S., D’Ambra V., Tabony M., Woods T.C., Hou X., Li Z. (2018). SM22alpha (Smooth Muscle protein 22-alpha) promoter-driven IGF1R (Insulin-like Growth Factor 1 Receptor) deficiency promotes atherosclerosis. Arterioscler. Thromb. Vasc. Biol..

[98] Shapiro L., Elsangeedy E., Lee H., Atala A., Yoo J.J., Lee S.J., Ju Y.M. (2019). In vitro evaluation of functionalized decellularized muscle scaffold for in situ skeletal muscle regeneration. Biomed. Mater..

[99] Manzella L., Massimino M., Stella S., Tirro E., Pennisi M.S., Martorana F., Motta G., Vitale S.R., Puma A., Romano C. (2019). Activation of the IGF axis in thyroid cancer: Implications for tumorigenesis and treatment. Int. J. Mol. Sci..

[100] Maffezzoni F., Formenti A.M. (2018). Acromegaly and bone. Minerva Endocrinol..

[101] Van Nieuwpoort I.C., Vlot M.C., Schaap L.A., Lips P., Drent M.L. (2018). The relationship between serum IGF-1, handgrip strength, physical performance and falls in elderly men and women. Eur. J. Endocrinol..

[102] Procaccini C., Santopaolo M., Faicchia D., Colamatteo A., Formisano L., de Candia P., Galgani M., De Rosa V., Matarese G. (2016). Role of metabolism in neurodegenerative disorders. Metabolism.

[103] Kopczak A., Stalla G.K., Uhr M., Lucae S., Hennings J., Ising M., Holsboer F., Kloiber S. (2015). IGF-I in major depression and antidepressant treatment response. Eur. Neuropsychopharmacol..

[104] Licata L., Lo Surdo P., Iannuccelli M., Palma A., Micarelli E., Perfetto L., Peluso D., Calderone A., Castagnoli L., Cesareni G. (2020). SIGNOR 2.0, the SIGnaling network open resource 2.0: 2019 update. Nucleic Acid. Res..

[105] Szklarczyk D., Gable A.L., Lyon D., Junge A., Wyder S., Huerta-Cepas J., Simonovic M., Doncheva N.T., Morris J.H., Bork P. (2019). STRING v11: Protein-protein association networks with increased coverage, supporting functional discovery in genome-wide experimental datasets. Nucleic Acid. Res..

[106] Bentzinger C.F., Wang Y.X., Rudnicki M.A. (2012). Building muscle: Molecular regulation of myogenesis. Cold Spring Harb. Perspect. Biol..

[107] Buckingham M., Relaix F. (2015). PAX3 and PAX7 as upstream regulators of myogenesis. Semin. Cell Dev. Biol..

[108] Forcina L., Miano C., Scicchitano B.M., Musaro A. (2019). Signals from the niche: Insights into the role of IGF-1 and IL-6 in modulating skeletal muscle fibrosis. Cells.

[109] Ding H., Wu T. (2018). Insulin-like growth factor binding proteins in autoimmune diseases. Front. Endocrinol..

[110] Boisclair Y.R., Rhoads R.P., Ueki I., Wang J., Ooi G.T. (2001). The acid-labile subunit (ALS) of the 150 kDa IGF-binding protein complex: An important but forgotten component of the circulating IGF system. J. Endocrinol..

[111] Tzeng H.E., Chen J.C., Tsai C.H., Kuo C.C., Hsu H.C., Hwang W.L., Fong Y.C., Tang C.H. (2011). CCN3 increases cell motility and MMP-13 expression in human chondrosarcoma through integrin-dependent pathway. J. Cell. Physiol..

[112] Bidlingmaier M., Strasburger C.J. (2010). Growth hormone. Handb. Exp. Pharmacol..

[113] Jones B.H., Hauschild V.D., Canham-Chervak M. (2018). Musculoskeletal training injury prevention in the U.S. Army: Evolution of the science and the public health approach. J. Sci. Med. Sport.

[114] Martins K.J., Gehrig S.M., Naim T., Saenger S., Baum D., Metzger F., Lynch G.S. (2013). Intramuscular administration of PEGylated IGF-I improves skeletal muscle regeneration after myotoxic injury. Growth Horm. IGF Res..

